# A Severe Acute Respiratory Syndrome extranet: supporting local communication and information dissemination

**DOI:** 10.1186/1472-6947-5-17

**Published:** 2005-06-20

**Authors:** Ruta K Valaitis, Noori Akhtar-Danesh, Cathy M Kealey, Glenn M Brunetti, Helen Thomas

**Affiliations:** 1 Public Health Research and Development Program, Public Health and Community Services Department, 1 Hughson St N, Hamilton, L8R 3L5, Canada; 2 Public Health and Community Services Department, Hamilton, L8R 3L5, Canada; 3 School of Nursing, McMaster University, 1200 Main Street West, Hamilton, L8N 3Z5, Canada

## Abstract

**Background:**

The objective of this study was to explore the use and perceptions of a local Severe Acute Respiratory Syndrome (SARS) Extranet and its potential to support future information and communication applications. The SARS Extranet was a single, managed electronic and limited access system to manage local, provincial and other SARS control information.

**Methods:**

During July, 2003, a web-based and paper-based survey was conducted with 53 SARS Steering Committee members in Hamilton. It assessed the use and perceptions of the Extranet that had been built to support the committee during the SARS outbreak. Before distribution, the survey was user-tested based on a think-aloud protocol, and revisions were made. Quantitative and qualitative questions were asked related to frequency of use of the Extranet, perceived overall usefulness of the resource, rationale for use, potential barriers, strengths and limitations, and potential future uses of the Extranet.

**Results:**

The response rate was 69.4% (n = 34). Of all respondents, 30 (88.2%) reported that they had visited the site, and rated it highly overall (mean = 4.0; 1 = low to 5 = high). However, the site was rated 3.4 compared with other communications strategies used during the outbreak. Almost half of all respondents (44.1%) visited the site at least once every few days. The two most common reasons the 30 respondents visited the Extranet were to access SARS Steering Committee minutes (63.3%) and to access Hamilton medical advisories (53.3%). The most commonly cited potential future uses for the Extranet were the sending of private emails to public health experts (63.3%), and surveillance (63.3%). No one encountered personal barriers in his or her use of the site, but several mentioned that time and duplication of email information were challenges.

**Conclusion:**

Despite higher rankings of various communication strategies during the SARS outbreak, such as email, meetings, teleconferences, and other web sites, users generally perceived a **local **Extranet as a useful support for the dissemination of **local **information during public health emergencies.

## Background

An Extranet is a private network that uses the Internet to securely share information or operations with selected partners; it is accessible from any web browser. The literature describes the use and potential benefits of local area Extranets in hospitals,[1] physician offices,[2,3] and small managed health care organizations [4]. A current U.S. National Emergency Medical Extranet (NEME) is being constructed to support emergency medicine and public health [5]. Building on the Centre for Disease Control's (CDC) national communication infrastructure, the US has begun to develop their Extranet to manage local public health emergencies, and plans to expand it for communicable disease surveillance and control activities [6].

A systematic review of the use of information technology in the event of bioterrorism evaluated web-based communication systems that link public health officials with clinicians and the public [7]. Although such systems have not been tested in crisis situations, the review indicates that they can securely manage disease-reporting needs of local and state officials. The systems evaluated most were those communicating abnormal findings in electronic medical records between institutions and clinicians. Three communication systems had the capacity to support rapid reporting and dissemination of information related to naturally occurring and bioterrorist-related infectious disease, although they required the use of electronic medical records.

A provincial Severe Acute Respiratory Syndrome (SARS) emergency was declared in Ontario on March 26, 2003. Two days later, a SARS Steering Committee was convened in Hamilton, Ontario, linking Public Health and Community Services (PHCS) with other partners (Table 1). Hamilton is located about 70 kilometers west of Toronto, which was the centre of the outbreak. Information management was critical during the crisis. Communication from the Ontario Provincial Operations Centre proceeded through email and fax to hospitals, long-term care facilities, health units, and the Ontario Medical Association. Simultaneously, information came from the World Health Organization (WHO), Health Canada, other health units and health care partners. The multi-source nature of the information made it difficult to manage and synthesize the most current, accurate information in a timely fashion. In addition, information impacting health units and Emergency Medical Services (EMS) might have reached only hospital staff. Directives changed often, necessitating version control measures to ensure that everyone was working from the most recent documents. This situation was difficult to manage with emails as updates were often sent daily and document file sizes were too large to send. A decision was made to develop a single, limited-access web-based system to manage constantly changing local and provincial SARS control information.

**Table 1:** Respondents by Agency or Institution (n = 34)

**Agency or institution**	**Frequency**	**Percentage**
Hospitals	11	32.4
Public Health and Community Service	10	29.4
Ontario Ministry of Health and Long-term Care (MOHLTC)	5	14.7
Emergency services	4	11.8
Community Care Access Centre	3	8.8
Other	1	2.9

**Total**	**34**	**100.0**

In response to demands from the steering committee for up-to-date information, the City of Hamilton's Public Health and Community Services (PHCS) and Information Technology (IT) Division developed and launched an Extranet on April 3, 2003. The Extranet was developed to support the SARS steering committee in disseminating to partners comprehensive information that was timely, current, accurate and relevant. The Extranet also supported the implementation of local strategies using a secure accessible and managed centralized access point. SARS steering committee members (N = 53) received a common ID and password to access the site. The site did not require users to connect through a Virtual Private Network (VPN), nor was there any validation of IP addresses to validate users. The Extranet, however, was viewed as a private network using the Internet to securely share information with partners. The security aspects made it an Extranet.

The secure site provided document distribution, version control of documents such as clinical guidelines and directives, and control of links to information for schools and workplaces. An email link was provided to access the health unit's web master. Because of the speed at which this site had to be developed, there was insufficient time for developing other interactive communication tools or tracking IP addresses. Ideally, participatory design principles, [8,9] would have been used to design the site, but there was no time to actively involve end users or track the costs to set up and maintain the site. Set-up and maintenance of the site was estimated to require one programmer (100 hours @ $40/hr), one Information Architect and Business/Technical Liaison (95 hours @ $50/hr), and one communicable disease expert (10 hours @ $75/hr) over the twelve weeks the Extranet was in use (total = $9,500).

The objectives of this research were to examine a) the use and perceptions of a local SARS Extranet, and b) its potential to support future public health information dissemination and communication with the local community.

## Methods

The local university ethics review board approved the study. Participants included all 53 SARS Steering Committee members. The authors designed and tested a survey containing 13 forced-choice and 8 open-ended questions, which focused on perceptions on the Extranet and on other SARS information and communication sources. Both web-based and paper-based surveys were used to ease completion and simplify data collection as well as to increase the response rate.

User testing was conducted with the online version of the survey to identify problems with use, ensure survey comprehension, calculate average completion time (10–15 minutes), and identify if dynamic or technical functionality posed challenges. A three-member observation team tested the web survey with four PHCS employees who were familiar with the SARS Extranet and comfortable using the web. Instructions were given to each participant based on a think-aloud protocol, [10-14]. Participants were asked to complete the web survey, while observers took notes. Participants were instructed to think out loud, make statements, or ask questions as they made their way through the survey. They were informed that observers would not respond, but that observers would benefit from hearing their comments and watching their actions. Observers noted problems spoken and/or observed, which were later discussed in a debriefing session after each test. They participated in a final debriefing session to identify problems that occurred with more than one participant; this process provided direction for survey improvements. Several minor survey modifications were made prior to the launch. The online survey form was dynamic and, depending on how a respondent replied to one question, determined the next series of questions. Thus, changes identified through the think-aloud technique related to branching of questions (If yes, go here; If no, go there). No other significant issues were identified. The paper-based survey was also modified slightly to match the web version.

On July 3^rd ^2003, all steering committee members were sent an information email along with a letter of introduction, an invitation to participate, and a direct link to the online survey. One week later, a reminder email was sent to non-respondents; two weeks later, the paper-based survey and self-addressed stamped envelope was mailed. Quantitative data were entered into SPSS for analysis. Two researchers independently coded the open-ended questions. Consensus was reached with the entire research team whenever differences in coding existed.

## Results

Of the 53 SARS Steering Committee members, four were ineligible as follows: one person moved, one was a research team member, and two were not active on the committee. Of the 49 eligible participants, three refused to complete the survey, three of them were disqualified because their responses were inconsistent, and nine of them did not respond. Respondents were representative of the steering committee, which included members from various agencies (Table 1). The response rate was 69.4% (n = 34).

### Hamilton SARS Extranet compared with other SARS communication strategies

Thirty-four respondents rated the usefulness (1-lowest to 5-highest) of communication strategies used during the outbreak (Table 2). Email, teleconferences, and face-to-face meetings were rated the highest. Among SARS web-based resources, the Ontario Ministry of Health and Long-Term Care (MOHLTC) (mean = 4.1), the Center for Disease Control (CDC) (mean = 4.0), and the World Health Organization (WHO) (mean = 4.0) sites were the most valued. The Hamilton SARS Extranet site was rated fifth among the web resources (mean = 3.4).

**Table 2:** Usefulness of Various Communication Strategies during the SARS outbreak to respondents (N = 34) (1= not at all useful to 5 = extremely useful)

**Communication strategy**	**N**	**Mean**	**SD**
E-mail	34	4.4	0.8
Teleconference	33	4.3	0.8
Face-to-face meeting	31	4.2	1.0
Ontario MOHLTC SARS private website	28	4.1	0.9
Centre for Disease Control website	24	4.0	1.0
WHO SARS website	24	4.0	1.2
Health Canada	28	3.9	1.2
City of Hamilton's SARS Extranet	28	3.4	1.2
Fax	23	3.3	1.1
City of Toronto Public Health Department's website	20	3.3	1.3

### Use and perceived utility of the Hamilton SARS extranet

The site had 3478 hits, and 30 respondents (88.2%) visited the Extranet (Table 3). The home page received the average number of 37.4 hits per day, while clinical guidelines and directives (10.3) and SARS Steering Committee minutes (7.3) received the next-highest average number of hits per day. Twenty-eight of them rated the quality of the Extranet and reported frequency of use. Even though the Extranet was rated lower than some other SARS web sites, when participants were asked specifically about the Hamilton Extranet, they rated it high overall (Mean = 4.0; SD = 0.8) (1 – lowest to 5 – highest). Criteria included relevance (Mean = 4.4; SD = 0.7), comprehensiveness (Mean = 4.0; SD = 0.8) accuracy (Mean = 4.3; SD = 0.6), and timeliness (Mean = 3.9; SD = 0.9). Most respondents (63.3%; n = 19) who used the Extranet visited the site at least once every few days. There was a difference in visiting habit between those who rated the Extranet most favourably (mean value ≥ 4 out of five points) and others. Those rating it most favourably visited the site more often (not statistically significant using two-sided Fisher's exact test, P > 0.50) than the others.

**Table 3:** Analysis of Hits to Pages on Site

**Page**	**Total hits**	**Average hits per day**
Clinical Guidelines and Directives	954	10.3
SARS Steering Committee minutes	683	7.3
Directives and Advisories	316	3.4
Resources for other professionals	187	2.0
Epidemiology	184	2.0
Schools/Workplace	171	1.8
Health Services	57	0.6
Hamilton Medical Advisories	42	0.5

Thirty participants were asked a forced-choice question about whether they visited the site and why they visited. Most commonly, users visited to obtain SARS Steering Committee minutes (63.3%), access "Hamilton medical advisories" (53.3%), and obtain the SARS Steering Committee contact list (53.3%) (Table 4). These results corroborate the number of actual hits per page, Table 3. Other reasons for visiting included getting access to provincial directives and links to other websites. Although the difference was only marginally significant (P = 0.063), those who rated the website most favorably cited more reasons for using it than did others.

**Table 4:** Extranet Resources Used by Number of Extranet Users (N = 30)

**Reason**	**Frequency**	**Percentage**
Steering committee minutes	19	63.3
Hamilton medical advisories	16	53.3
Steering committee contact list	16	53.3
Provincial directives	14	46.7
Links to other websites	12	40.0
Emergency telephone numbers	10	33.3
Clinical guidelines	8	26.7
SARS signs	5	16.7
SARS assessment clinic information	5	16.7
Resources for other health professionals	5	16.7
Link SARS private website: Ontario Ministry of Health and Long-Term Care	2	6.7
Other	3	10.0

Eighteen participants revealed that they shared information from the Extranet with others outside of the committee. The information they shared most often included provincial directives and local information such as Hamilton medical advisories and Steering Committee minutes (Table 5). The average number of hits per page corroborates this finding, as these resources were used the most (Table 4). Eight (22.8%) respondents reported sharing the website address, username and password with middle management and staff.

**Table 5:** Type of Information Shared by those who Shared Information with People Outside the SARS Steering Committee (N = 18)

**Information**	**Frequency**	**Percentage**
Provincial directives clinical guidelines	10	55.6
Hamilton medical advisories	9	50.0
Steering committee minutes	8	44.4
Resources for other health professionals	7	38.9
SARS signs	6	33.3
Steering committee contact list	4	22.2
SARS assessment clinic information	3	16.7
Website/ Web address	3	16.7
Emergency telephone numbers	3	16.7

Open-ended questions were included to gain a better understanding of the barriers, strengths, limitations, and other features of a local Extranet. Most respondents indicated that they experienced no personal barriers to accessing the Extranet. However, a few respondents indicated that time to access the site and duplication of information sent through email and the Extranet were barriers. A concern was raised related to Extranet access: "Some folks had access/password who should not have access, and were following information not yet implemented or disseminated internally." One person indicated that access was expanded to others as the outbreak was prolonged.

Regarding the strengths of the system, many respondents indicated that they appreciated the ability to access current, timely and comprehensive information in one location. One respondent noted " [It was] very useful for staff to have info in one place, in early days of SARS where no other provincial web sites [were] set up to access information." Another participant noted that the Extranet was able to "dispel rumours [and] contained a broad scope of information in one locale."

Respondents identified no weaknesses of the Extranet; however, a few commented on the manpower and time needed to keep the site current as well as the reliance on IT staff to upload information in a timely fashion. A few also noted the redundancy of the site; for example, "Once the provincial site [was] up [there was] no need for the Extranet." One participant highlighted a problem with Internet access: " [The] web network went down on one occasion during [the] crisis and delayed [the] info getting out."

Respondents mentioned that using an electronic system for communication during an emergency was a good learning experience. Some noted areas for improvement: They wanted technical support to be available (help-desk) for participants who might have difficulties accessing the secure web site. They also identified a need for a system that can be activated immediately in future public health emergencies.

### Potential future use of a local Extranet

Thirty respondents identified features they would like to see on a future Extranet so as to enhance communication with the public health department. Of these, 19 (63.3%) wanted to use the Extranet as a mechanism to send private email to public health experts; 19 (63.3%) wanted to use it to share a database for surveillance of infectious diseases; 15 (50%) wanted to use it as a bulletin board communication system; 9 (30.0%) wanted online certificate courses on public health matters; and 1 (3.3%) wanted to use it as a mechanism for physicians to send immunization data. From the open-ended questions, eleven respondents mentioned that the Extranet could be used for communicable disease communication, surveillance and/or reporting, and two respondents believed that the Extranet could support preparedness for future public health emergencies.

## Discussion

Our results showed that participants preferred email, face-to-face meetings, or phone communication to any Internet communication strategy. This is not surprising, considering these strategies are more interactive than visiting a typical web site. Respondents indicated that they wanted future Extranets to support private email to experts as well as have a bulletin board for feature use, validating our conjecture that they would prefer more interactivity. Two-way communication features may be important features to consider in the creation of future Extranet solutions so as to enhance interactivity. Further research is needed to measure the perceived value and impact of interactive technologies. In addition to having synchronous communication modes, such as a discussion board or email link, web-conferencing and real-time online communication using text chat may provide more effective interactive web-based solutions.

We can speculate on the various reasons why email was a preferred method of communication compared with web sites. An administrator likely views the use of email as part of his or her daily routine activity, but does not view searching web sites as a routine. Information is automatically 'pushed' to users in email, whereas users need to actively seek information from an Internet site. Furthermore, a web site is typically less interactive than email, with which users can easily reply to senders. Although the Hamilton Extranet had an email link, it was not used. The Extranet was likely not perceived by users as a two-way communication tool but, rather, a place to find information.

The Hamilton Extranet was rated low relative to other SARS web sites. Perhaps this was because the newly developed Hamilton Extranet had not had a chance to establish its credibility compared with long-established sites such as those from the CDC, WHO and the provincial MoHLTC. Despite this finding, the majority rated the Hamilton Extranet high overall, considering its own merits. Possible reasons for this positive opinion may be extrapolated from the findings: Given the most common reasons for visiting the site, the Extranet was perceived as useful to support local information needs such as meeting minutes, and Hamilton directives. In addition, until the provincial SARS site was launched, the Hamilton Extranet was the only source of provincial information. Once the MOHLTC website was launched, redundancy with Extranet information was inevitable; our findings supported this view. Also, provincial MoHLTC staff may not have been interested in specific local issues.

Despite the Extranet's shortcomings, the findings generally support the view that access to both local and provincial information is needed; however, better planning, coordination and alignment of local and provincial web communication strategies are required. It is essential to clarify without delay where the responsibility for communication begins and ends among local, provincial and federal public health agencies during emergencies.

The development of a local Extranet must be viewed as an evolutionary process. SARS Extranet users were moving away from email and fax communication to rely more on the Internet. The Extranet was built quickly to meet immediate local needs during the SARS emergency without taking time to fully engage end-users in design. Governance structures are needed to set guidelines about the practice of sharing passwords. Health units are thus urged to work with their communities before the next emergency to create a secure password-protected Internet communication system. Through such partnerships, local communities will feel more ownership and familiarity with the communication infrastructure.

Two areas of concern for a future Extranet are the security of the site and privacy of information. Many users shared with others both the information and the user IDs and passwords. Had there been sensitive information on the Extranet, a risk of improper access or release of information would have existed. These issues can be dealt with by more stringent security policies and technological improvements to the Extranet.

Looking beyond SARS, respondents were asked to respond to potential future Extranet applications for public health: Communication on communicable disease was the most common response. Not surprisingly, this response reflects the needs of the SARS Steering Committee, which was responsible for infection control during the SARS emergency. An Extranet also has the potential to include activities such as surveillance and public health education. In addition, an Extranet for use during non-emergency situations may create a useful infrastructure to support local communication in future emergencies.

This study has some important limitations. The survey was conducted three months after the initial SARS outbreak, so participants may not have accurately recalled their use of the site. This uncertainty is further complicated by the wide variety of SARS sites. The lack of statistical significance may be a result of the small size of the respondents. Finally, because the Extranet was developed quickly, it was not possible to plan for a rigorous evaluation. Qualitative evaluation methods would have been useful to answer questions about why participants rated the Hamilton site lower than other sites. Given more time for planning, set-up, and design of the Extranet, long-and short-term objectives would have been developed from which to frame a program evaluation. A more accurate system for tracking individual use would also have been incorporated. Better assessment of the human and financial costs of running the Extranet would have been a useful measure against the perceived value of the Extranet.

## Conclusion

In conclusion, our study showed that interactive communication strategies – email, face-to-face meetings and teleconferences – were preferred over static web sites during the SARS outbreak. This finding has important implications for the future development and evaluation of web-based communication solutions. It is likely that more interactivity in web-based communication solutions would enhance their use and value. Although the CDC, WHO, and federal and provincial sources of SARS health information on the Internet were rated higher than that on the Hamilton Extranet, a local area Extranet appears to have an important role in supporting **local **information sharing and communication in an emergency situation. There is a need, however, for anticipatory planning, coordination and development of an Extranet communication system to ensure we are prepared for the next public health emergency. In addition, the findings indicate that users envisioned an expanded role of a local Extranet to support public health communication and education beyond the management of emergencies. Lessons learned from this study provide a foundation on which to build for future emergencies.

## Competing interests

The authors have no competing financial interests with regard to any aspect of this work. Two of the authors (CK and GB) were involved in the development of the site and participated in various aspects of the research work (see authors contributions for details).

## Authors' contributions

RV and HT conceived of the study and the design; all the authors, led by RV and HT, participated in the creation of the data collection tool. Data collection and entry was led by RV and NA. Data analysis of open-ended questions was conducted primarily by RV, HT and NA; however, CK and GB assisted in the interpretation of the results because they had a better understanding of the context of some participants' comments, having been involved in the development and maintenance of the site. Statistical analysis was conducted by NA. All the authors assisted in drafting the article and making edits, and they all contributed to the discussion and conclusion.

## Pre-publication history

The pre-publication history for this paper can be accessed here:



## References

[B1] DeLeonardis R, Sansotta C, Ferlazzo M, Vermigloi G, Faranda C (2002). Wired and wireless network solution for the integrated management of data and images. Radiologia Medica.

[B2] Bero CL, Glaser J, Franklin J (2003). Partners Community HealthCare Extranet (PCHInet): a business plan. Journal of Healthcare Information Management.

[B3] Chin TL (1998). MedPartners extranet survives. Health Data Management.

[B4] Meyeroff WJ, Meyeroff RE (1998). Got it? Share it. No managed care organization is too small for an extranet to pay back big. HealthCare Informatics.

[B5] Barthell EN, Pemble KR (2003). The National Emergency Medical Extranet project. Journal of Emergency Medicine.

[B6] Doniger AS, Labowitz D, Mershon S, Gotham IJ (2001). Design and implementation of a local Health Alert Network. Journal of Public Health Management & Practice.

[B7] Owens D (2002). Bioterrorism Preparedness and Response: Use of Information Technologies and Decision Support Systems.

[B8] Bodker S, Gronbaek K, Kyng M, Baecker RM, Grudin J, Buxton WA and Greenberg JS (1995). Cooperative design:techniques and experiences from the Scandanavian Scene. Human Computer Interaction: Toward the Year 2000.

[B9] Ellis RD, Jankowski TB, Jasper JE (1998). Participatory design of an Internet-based information system for aging services professionals. The Gerontologist.

[B10] Boren M, Ramey J (2000). Thinking aloud: Reconciling theory and practice. IEEE Transactions on Professional Communication.

[B11] Hoppe M, Wells E, Morrison D, Wilsdon A, Gillmore (1995). Using focus groups to discuss sensitive topics with children. Evaluation Review.

[B12] Hughes J, Parkes S (2003). Trends in the use of verbal protocol analysis in software engeneering research. Behavoiur and Information Technology.

[B13] Branch J (2000). Investigating the information-seeking processes of adolescents: The value of using think alouds and think afters. Library and Information Science Research.

[B14] Van Waes L (2000). Thinking aloud as a method for testing the useability of websites: The influence of task variation on the evaluation of hypeertext. IEEE Transactions on Professional Communication.

